# Timing and type of breast reconstruction in SweBRO 3: long-term outcomes

**DOI:** 10.1093/bjs/znae240

**Published:** 2024-09-24

**Authors:** Rojda Gümüscü, Fredrik Wärnberg, Jana de Boniface, Malin Sund, Kristina Åhsberg, Emma Hansson, Folke Folkvaljon, Dmytro Unukovych, Maria Mani

**Affiliations:** Department of Surgical Sciences, Uppsala University, Uppsala, Sweden; Department of Surgical Sciences, Uppsala University, Uppsala, Sweden; Department of Surgery, Sahlgrenska University Hospital, Institute of Clinical Sciences, Sahlgrenska Academy at Gothenburg University, Gothenburg, Sweden; Department of Surgery, Capio St. Göran’s Hospital, Stockholm, Sweden; Department of Medicine and Surgery, Karolinska Intitutet, Stockholm, Sweden; Department of Surgery and Perioperative Sciences, Umeå University, Umeå, Sweden; Department of Surgery, University of Helsinki and Helsinki University Hospital, Helsinki, Finland; Department of Surgery, Halland Hospital, Halmstad, Sweden; Department of Clinical Sciences, Lund University, Lund, Sweden; Department of Plastic Surgery, Institute of Clinical Sciences, Sahlgrenska Academy, University of Gothenburg, Gothenburg, Sweden; Region Västra Götaland, Sahlgrenska University Hospital, Department of Plastic and Reconstructive Surgery, Gothenburg, Sweden; Sveastat AB, Kungsbacka, Sweden; Department of Plastic and Craniofacial Surgery, Karolinska University Hospital, Stockholm, Sweden; Department of Surgical Sciences, Uppsala University, Uppsala, Sweden; Department of Plastic and Reconstructive Surgery, Uppsala University Hospital, Uppsala, Sweden

## Abstract

**Background:**

Breast reconstruction after mastectomy helps women with breast cancer feel better about their bodies and lives. There is debate about the best time and type of reconstruction (immediate *versus* delayed, and using own tissue *versus* implants). Long-term studies are rare.

**Aim:**

This study looked at long-term results of different breast reconstruction methods and timings in Swedish women who had mastectomies in 2000, 2005 or 2010. It focused on how satisfied the women were with their surgeries and their quality of life.

**Method:**

The study included 5853 women from the Swedish National Breast Cancer Registry who had mastectomies in 2000, 2005 or 2010. Of these, 2904 women answered the survey, and 895 had breast reconstruction. Satisfaction and quality of life were measured using two surveys: EORTC QLQ-BRECON23 and BREAST-Q.

**Results:**

Of the women who answered the survey, 895 (31%) had breast reconstruction. Of these, 176 (20%) had immediate reconstruction, and 719 (80%) had delayed reconstruction; 58% had implant-based reconstructions, 31% had reconstructions using their own tissue, 2% had both types and 9% did not report the type of reconstruction. There were no major differences in satisfaction between immediate and delayed reconstruction. Women who used their own tissue were more satisfied with their results and breast appearance than those with implants.

**Conclusion:**

Autologous reconstruction leads to better satisfaction and outcomes than implants. The timing of reconstruction (immediate versus delayed) was less of an influence on quality of life.

## Teaser

Breast reconstruction following mastectomy plays a crucial role in restoring body image and quality of life for women with breast cancer. The optimal timing and reconstruction type remain subjects of debate, and population-based long-term studies on this topic are lacking. This national long-term follow-up survey highlights better well-being and quality of life among women with autologous reconstructions compared to implant-based reconstructions.

## Introduction

Breast cancer remains a significant global health burden affecting millions of women, and its management sometimes involves mastectomy, a procedure that can significantly impact physical and emotional well-being^1,2^. In the pursuit of restoring physical appearance and enhancing the health-related quality of life (HRQoL), breast reconstruction has emerged as a vital component of the treatment process^2^.

The timing and method of breast reconstruction can vary, with two primary approaches commonly used; that is, immediate *versus* delayed reconstruction and autologous *versus* implant-based reconstruction. The choice of reconstruction timing and method depends on several factors, including the patient’s overall health, cancer treatment plan, body type and the personal preferences of the patient and surgeon. Such preferences, however, vary considerably and the scientific evidence for when and how to reconstruct in order to obtain the best long-term results is low.

Immediate breast reconstruction (IBR) is performed at the same time as the mastectomy. This approach has the advantage of never living without a breast, which can help alleviate the emotional impact of a breast loss. Delayed reconstruction (DBR) is performed months or years after the mastectomy. It allows the patient to recover from the necessary treatments and ensure cancer control before undergoing reconstruction.

A previous study has shown no significant differences in patient satisfaction, psychosocial, sexual or physical well-being between IBR and DBR, despite being based on a prospective multicentre study with a response rate exceeding 91% and a 2-year follow-up period^3^.

A review of nine studies showed that IBR had a positive impact on patient well-being although the findings are constrained by variables such as small study sizes and inconsistent methodologies. Psychological support was suggested to be crucial and should be available for all breast cancer surgery patients due to the profound emotional effects^4^. However, there was an advantage of choosing IBR with better psychological well-being.

Other findings include improved body image in IBR patients according to Harcourt and Rumsey^5^ and better psychosocial well- being in IBR patients reported by Al Ghazal *et al.*^3^. Harcourt and Rumsey^5^, however, suggested that the surgical procedure itself, regardless of reconstruction, did not significantly impact body image.

Implant-based reconstruction involves the use of breast implants or expandable implants to recreate the breast mound. Autologous reconstruction, also known as flap reconstruction, employs a woman's own tissue to reconstruct the breast. Depending on patient anatomy, the tissue can usually be taken from the abdomen (for example, deep inferior epigastric perforator, DIEP flap), back (for example, latissimus dorsi, LD flap), or thigh/gluteal area (for example, profunda artery perforator, PAP flap). These methods generally lead to a breast that appears and feels more natural. In some cases, a combination of both implant and autologous tissue is used.

Although the existing literature highlights enhanced long-term durability, decreased rates of reconstructive failure and lower occurrences of surgical site infections associated with autologous tissue^6^, implant-based reconstruction offers a shorter recovery period and avoids donor-site morbidity^6–9^.

Patient-reported outcome measures (PROMs)^8,9^ have become an independent parameter for treatment outcomes alongside clinical measurements^10,11^. There are few systematic reviews analysing PROMs in autologous *versus* implant-based reconstruction, hindering the determination of a patient-preferred technique. The largest systematic review by Phan *et al*.^12^ including 13 published articles showed that the autologous group, assessed by the BREAST-Q, demonstrated higher satisfaction levels in both breast-related and psychosocial well-being compared to implant-based reconstruction. There were less pronounced differences in physical well-being, and the least significant variance was observed in sexual well-being. Similar trends were identified with another validated questionnaire the EORTC-QLQ-BR23/C30.

The primary objective of the current study is to evaluate the differences in long-term outcomes in HRQoL between immediate and delayed breast reconstruction, as well as to identify patient-reported differences between autologous and implant-based procedures in long-term.

## Methods

### Study design and protocol

The Swedish Breast Reconstruction Outcome Study (SweBRO study) was designed to assess the nationwide distribution of postmastectomy breast reconstruction. Its primary objective is to examine variations in patient well-being and quality of life across different demographic groups. Unukovych *et al.*^13^ presented the first part, SweBRO 1, which explored the demographics and patterns across Sweden. In the second part of the study Gümüscü *et al*. focused on analysing the differences between two distinct groups of mastectomy alone *versus* mastectomy and breast reconstruction^6^. The present third part provides subgroup analyses to identify potential differences in patient well-being associated with reconstruction methods and timing of breast reconstruction.

### Study population and setting

The survey was conducted between 20 April 2016 and 22 September 2016 in Sweden. The study population consisted of women who had been diagnosed with breast cancer and had undergone a mastectomy in the years 2000, 2005 or 2010. The selection of these specific years allowed for the assessment of patients at 5, 10 and 15 years after their mastectomy procedures. To ensure comprehensive coverage, the study utilized data from the National Breast Cancer Registry, with a 99% coverage rate^8^. The National Cause of Death Registry was cross-referenced to exclude deceased individuals from the potential study participants.

### Clinical data collection

Data regarding tumour characteristics and treatment details, including surgical interventions, chemotherapy, radiotherapy and endocrine treatment, were retrieved from the National Breast Cancer Registry. In addition to clinical data, the survey included self-reported information on sociodemographic characteristics, healthcare services availed and the type of breast reconstructive surgery pursued by the patients. Breast reconstruction information was based entirely on patient reports 5, 10 or 15 years after mastectomy. The timing was defined as ‘immediate’ if the patient reported that mastectomy and reconstruction were done simultaneously, and was defined as ‘delayed’ otherwise. Patients reported their reconstruction to be either ‘implant-based’, ‘autologous’ or a combination of those.

Invitation letters were sent out to all identified individuals. The survey was designed to offer participants the choice to respond either on paper or online, with all survey questions provided in Swedish. The survey process was previously described in detail^13^ ensuring that all relevant aspects were adequately addressed.

### Patient-reported outcome measurements

The survey included validated PROMs to assess the participants’ health-related quality of life and well-being. The specific PROMs used in this study were the BREAST-Q and the EORTC QLQ-BRECON23 reconstruction postoperative module questionnaires. Both questionnaires are widely used in clinical research and enable the collection of standardized and meaningful data from breast cancer patients operated with mastectomy and reconstruction. By incorporating patient perspectives and experiences, these questionnaires contribute to advancing medical knowledge and refining breast cancer treatment protocols, ultimately leading to better patient outcomes and HRQoL^7^.

EORTC QLQ-BRECON23 was developed by the European Organization for Research and Treatment of Cancer group to assess the specific impact of breast reconstruction after mastectomy^8^ covering aspects such as appearance, emotional well-being, satisfaction with the reconstructive process and the impact on daily life. The validated Swedish version was used in this study^9^.

BREAST-Q is a prevalent and validated PROM questionnaire designed to assess the quality of life and patient satisfaction related to various aspects of breast surgery and breast-related treatments^10,11^. Developed by a group at the Memorial Sloan Kettering Cancer Center, this questionnaire evaluates physical, psychosocial and sexual well-being following breast surgery focusing on breast appearance, satisfaction with the surgical outcome, impact on daily activities and emotional well-being. The Swedish version of Breast Q version 1.0 was used in the current study.

### Ethical approval

The study was approved by the regional ethics committee, Uppsala University (Dnr 2014/354 and Dnr 2014:354/1).

### Statistical analysis

Descriptive statistics for the study cohort and distribution of missing data have previously been reported^13^. Statistical hypothesis testing was done comparing survey responses by groups defined according to self-reported breast reconstruction status (immediate *versus* delayed and autologous *versus* implant-based). Due to the ordinal nature of the PROM scores and observed non-normality in their distribution, all comparisons were based on the Wilcoxon rank sum test of equal distribution between reconstructed and non-reconstructed women. No causality claims are made and reported values of *P* are only interpreted as indicating differences between the observed distributions of study data. The overall type I error (alpha) was controlled using an unweighted Bonferroni approach and was set to 0.05, split equally across four clusters defined by questionnaires: EORTC QLQ-C30, EORTC QLQ-BR23, EORTC QLQ-BRECON23 and BREAST-Q reconstruction postoperative module (*Fig. S1*). Of these, the current study focuses on the BRECON23 and BREAST-Q. Each questionnaire contains several domains and higher scores mean better HRQoL or higher satisfaction (in the BREAST-Q), and higher scores mean better higher satisfaction/functioning or worse symptoms (in the QLQ-BRECON23) (*Table S1*). Findings were analysed overall and in 16 prespecified subgroups, defined by demographics and baseline characteristics. Due to the alpha split, questionnaires with more domains to test had more stringent limits for statistical significance in each hypothesis test (lower *P* for statistical significance). The limit for statistical significance was hence set to *P* < 0.000040849 for BRECON23 and *P* < 0.000036764 for BREAST-Q. All tests were two-sided and R version 4.2.2 (R Foundation for Statistical Computing, Vienna, Austria) was used for all statistical analyses.

## Results

A total of 5853 women were eligible for inclusion in the study; 1259, 1976 and 2618 from years 2000, 2005 and 2010 respectively. In total, 2904 (50%) women responded to the questionnaires and of these 895 (31%) reported having undergone breast reconstruction (BR); of whom 176 of 895 (20%) women stated to have undergone immediate and 719 of 895 (80%) delayed BR (*Table 1*).

**Table 1:** From SweBRO 1

	Total (n)	Responded	Did not respond	*P*
**Women**	5853 (100)	2904 (100)	2949 (100)	
Selection year*				0.001
2000	1259 (22)	593 (20)	666 (23)	
2005	1976 (34)	943 (32)	1033 (35)	
2010	2618 (45)	1368 (47)	1250 (42)	
**Region of residence**				<0.001
Stockholm-Gotland	1109 (19)	591 (20)	518 (18)	
Uppsala-Örebro	1084 (19)	578 (20)	506 (17)	
Southeast	677 (12)	343 (12)	334 (11)	
South	1511 (26)	732 (25)	779 (26)	
West	1097 (19)	476 (16)	621 (21)	
North	375 (6)	184 (6)	191 (6)	
**Age at selection, years**				<0.001
Median (i.q.r.)	57 (49–65)	49 (44–56)	60 (53–68)	
<50	795 (27)	451 (50)	344 (17)	
50–59	893 (31)	309 (35)	584 (29)	
60–69	811 (28)	120 (13)	691 (34)	
>69	405 (14)	15 (2)	390 (19)	
**Age at survey, years**				<0.001
Median (i.q.r.)	69 (60–78)	67 (58–74)	73 (62–82)	
<50	412 (7)	221 (8)	191 (6)	
50–59	996 (17)	583 (20)	413 (14)	
60–69	1524 (26)	908 (31)	616 (21)	
>69	2921 (50)	1192 (41)	1729 (59)	
**Mastectomy side**				0.4
Right	2797 (48)	1368 (47)	1429 (48)	
Left	2970 (51)	1497 (52)	1473 (50)	
Bilateral	86 (1)	39 (1)	47 (2)	
**T stage**				<0.001
T0/Tis	778 (13)	423 (15)	355 (12)	
T1	1732 (30)	901 (31)	831 (28)	
T2	1889 (32)	910 (31)	979 (33)	
T3	340 (6)	175 (6)	165 (6)	
T4	63 (1)	21 (1)	42 (1)	
Tx/Missing data	1051 (18)	474 (16)	577 (20)	
**N stage**				<0.001
N0	3053 (52)	1576 (54)	1477 (50)	
N+	856 (15)	460 (16)	396 (13)	
Nx/Missing data	1944 (33)	868 (30)	1076 (36)	
**M stage**				0.002
Mx/M0	5120 (87)	2585 (89)	2535 (86)	
M1	17 (0)	7 (0)	10 (0)	
Missing data	716 (12)	312 (11)	404 (14)	
**Neoadjuvant therapy**				0.004
No neoadjuvant therapy	4593 (78)	2306 (79)	2287 (78)	
Received neoadjuvant therapy	330 (6)	179 (6)	151 (5)	
Missing data (pre-INCA in ‘Södra’)	930 (16)	419 (14)	511 (17)	
**Postoperative radiotherapy**				<0.001
No postoperative radiotherapy	3864 (66)	1815 (62)	2049 (69)	
Received postoperative radiotherapy	1989 (34)	1089 (38)	900 (31)	
**Postoperative endocrine therapy**				0.3
No postoperative endocrine therapy	3261 (56)	1637 (56)	1624 (55)	
Received postoperative endocrine therapy	2592 (44)	1267 (44)	1325 (45)	
**Postoperative chemotherapy**				<0.001
No postoperative chemotherapy	4391 (75)	2057 (71)	2334 (79)	
Received postoperative chemotherapy	1462 (25)	847 (29)	615 (21)	

Baseline characteristics and demographics of all women.

^*^Selection was done on year of diagnosis in all regions except for the ‘South’ region in which women were selected based on year of mastectomy.

Regarding reconstruction type, 516 (58%) stated being reconstructed with implants and 281 (31%) with autologous tissue. In 20 (2%) women a combination of techniques had been used and in 78 (9%) the technique was not reported.

### Immediate *versus* delayed breast reconstruction

The timing of breast reconstruction did not correlate with the PROM by either EORTC QLQ-BRECON23 or the BREAST-Q. In a single subgroup analysis of patients with no lymph node metastases (N0), the satisfaction with nipples was higher in women with delayed reconstruction compared to immediate, mean 57 *versus* 45.

### Autologous *versus* implant-based BR

#### EORTC QLQ-BRECON23

Overall (*n* = 797), women with autologous reconstruction were more satisfied with surgery (71 *versus* 58; 13 points difference) and reported higher levels of satisfaction with their breasts (63 *versus* 47; 16 points) (*Table 2*). These differences remained significant in favour of autologous reconstruction regardless of timepoints (years 2000, 2005, 2010), patient age (<50 and ≥50 years), radiotherapy (RT+ and RT–), chemotherapy (CT+ and CT–) and endocrine therapy (ET+ and ET–) (*Table 2*). The largest differences were seen in the subgroup among those operated in 2000, that is 15 years after mastectomy at the time of survey. The satisfaction with breast was 66 *versus* 42 (difference of 24 points) and satisfaction with surgery 75 *versus* 54 (21 points). Among patients who had been treated with radiotherapy (RT+), satisfaction with breast (70 *versus* 55; 25 points difference) and satisfaction with surgery (64 *versus* 45; 21 points) were also in favour of autologous reconstruction. The same pattern was seen for those with tumour stage T2–T4 where radiotherapy was regarded as a potential confounding factor (*Table 2*).

**Table 2:** BREAST-Q reconstruction postop module scores by breast reconstruction modality, only showing scales and subgroups with statistically significant differences between implant-based and autologous breast reconstruction

	Total	Implant-based	Autologous	*P*
**All women, *n***	797	516	281	
**BREAST-Q postop: Satisfaction with breasts**				
* Available (%)*	794 (100)	514 (100)	280 (100)	
Mean(s.d.)	59(18)	56(16)	67(18)	<0.000036764
**BREAST-Q postop: Satisfaction with surgeon**				
Available (%)	790 (99)	511 (99)	279 (99)	
Mean(s.d.)	82(20)	80(21)	87(18)	<0.000036764
**Subset: selected 2000, *n***	153	104	49	
**BREAST-Q postop: Satisfaction with breasts**				
Available (%)	153 (100)	104 (100)	49 (100)	
Mean(s.d.)	57(18)	52(18)	67(16)	<0.000036764
**Subset: selected 2005, n**	252	164	88	
**BREAST-Q postop: Satisfaction with breasts**				
Available (%)	249 (99)	162 (99)	87 (99)	
Mean(s.d.)	59(18)	54(16)	67(18)	<0.000036764
**Subset: selected 2010, *n***	392	248	144	
**BREAST-Q postop: Satisfaction with breasts**				
Available (%)	392 (100)	248 (100)	144 (100)	
Mean(s.d.)	61(17)	58(16)	66(18)	<0.000036764
**Subset: age <50 years at selection, *n***	411	255	156	
**BREAST-Q postop: Satisfaction with breasts**				
Available (%)	411 (100)	255 (100)	156 (100)	
Mean(s.d.)	60(17)	56(14)	67(18)	<0.000036764
**Subset: age ≥50 years at selection, *n***	386	261	125	
**BREAST-Q postop: Satisfaction with breasts**				
Available (%)	383 (99)	259 (99)	124 (99)	
Mean(s.d.)	59(19)	55(18)	66(18)	<0.000036764
**Subset: T2–T4, *n***	319	182	137	
**BREAST-Q postop: Satisfaction with breasts**				
Available (%)	318 (100)	181 (99)	137 (100)	
Mean(s.d.)	60(19)	54(18)	68(18)	<0.000036764
**Subset: N0, *n***	480	341	139	
**BREAST-Q postop: Satisfaction with breasts**				
Available (%)	479 (100)	340 (100)	139 (100)	
Mean(s.d.)	59(18)	56(16)	65(19)	<0.000036764
**BREAST-Q postop: Satisfaction with surgeon**				
Available (%)	476 (99)	337 (99)	139 (100)	
Mean(s.d.)	81(20)	79(20)	88(16)	<0.000036764
**Subset: RT–, n**	501	397	104	
**BREAST-Q postop: Satisfaction with breasts**				
Available (%)	499 (100)	396 (100)	103 (99)	
Mean(s.d.)	58(17)	56(16)	66(19)	<0.000036764
**Subset: RT+, *n***	296	119	177	
**BREAST-Q postop: Satisfaction with breasts**				
Available (%)	295 (100)	118 (99)	177 (100)	
Mean(s.d.)	62(18)	54(17)	67(17)	<0.000036764
**Subset: ET–, *n***	467	324	143	
**BREAST-Q postop: Satisfaction with breasts**				
Available (%)	465 (100)	323 (100)	142 (99)	
Mean(s.d.)	59(18)	55(16)	66(19)	<0.000036764
**Subset: ET+, *n***	330	192	138	
**BREAST-Q postop: Satisfaction with breasts**				
Available (%)	329 (100)	191 (99)	138 (100)	
Mean(s.d.)	60(18)	56(17)	67(17)	<0.000036764
**Subset: CT−, *n***	523	373	150	
**BREAST-Q postop: Satisfaction with breasts**				
Available (%)	522 (100)	373 (100)	149 (99)	
Mean(s.d.)	58(17)	55(16)	65(18)	<0.000036764
**Subset: CT+, *n***	274	143	131	
**BREAST-Q postop: Satisfaction with breasts**				
Available (%)	272 (99)	141 (99)	131 (100)	
Mean(s.d.)	63(18)	58(17)	68(18)	<0.000036764

In all BREAST-Q scales, higher scores are better. The survey was administered in 2015–2016, either 15 years (women selected in 2000), 10 years (women selected in 2005) or 5 years (women selected in 2010) after mastectomy. *P* are based on Wilcoxon rank sum test and are only calculated if there are at least three unique values in each group. Due to the Bonferroni adjustment, only *P*  *<* 0.000036764 are considered significant. CT, chemotherapy; ET, endocrine therapy; RT, radiotherapy.

#### BREAST-Q

BREAST-Q results for the whole study population also showed a higher level of satisfaction with breasts (67 *versus* 56; 11 points) and satisfaction with surgery (87 *versus* 80; 7 points) for those operated with autologous reconstruction (*Table 3*). The satisfaction with breasts remained significantly higher for autologous reconstruction at all time points (patients operated in 2000, 2005, 2010), regardless of patient age (<50 and ≥50 years), given radiotherapy (RT+ and RT−), chemotherapy (CT+ and CT−) and endocrine therapy (ET+ and ET−) (*Table 3*).

**Table 3:** QLQ-BRECON23 scores by breast reconstruction modality, only showing domains and subgroups with statistically significant differences between implant-based and autologous breast reconstruction

	Total	Implant-based	Autologous	*P*
**All women, *n***	797	516	281	
**QLQ-BRECON23: Satisfaction with breast cosmetic**				
Available (%)	787 (99)	510 (99)	277 (99)	
Mean(s.d.)	53(28)	47(27)	63(27)	<0.000040849
**QLQ-BRECON23: Satisfaction with surgery**				
Available (%)	743 (93)	476 (92)	267 (95)	
Mean(s.d.)	63(26)	58(26)	71(25)	<0.000040849
**Subset: selected 2000, *n***	153	104	49	
**QLQ-BRECON23: Satisfaction with breast cosmetic**				
Available (%)	150 (98)	102 (98)	48 (98)	
Mean(s.d.)	49(29)	42(28)	66(24)	<0.000040849
**QLQ-BRECON23: Satisfaction with surgery**				
Available (%)	144 (94)	96 (92)	48 (98)	
Mean(s.d.)	61(29)	54(29)	75(23)	<0.000040849
**Subset: selected 2005, *n***	252	164	88	
**QLQ-BRECON23: Satisfaction with breast cosmetic**				
Available (%)	246 (98)	161 (98)	85 (97)	
Mean(s.d.)	51(28)	45(27)	62(28)	<0.000040849
**QLQ-BRECON23: Satisfaction with surgery**				
Available (%)	232 (92)	150 (91)	82 (93)	
Mean(s.d.)	61(27)	56(26)	71(26)	<0.000040849
**Subset: selected 2010, *n***	392	248	144	
**QLQ-BRECON23: Satisfaction with breast cosmetic**				
Available (%)	391 (100)	247 (100)	144 (100)	
Mean(s.d.)	55(27)	50(26)	64(28)	<0.000040849
**QLQ-BRECON23: Treatment side effects**				
Available (%)	389 (99)	246 (99)	143 (99)	
Mean(s.d.)	11(19)	8(16)	17(23)	<0.000040849
**Subset: age <50 years at selection, *n***	411	255	156	
**QLQ-BRECON23: Satisfaction with breast cosmetic**				
Available (%)	410 (100)	254 (100)	156 (100)	
Mean(s.d.)	54(26)	48(25)	64(26)	<0.000040849
**QLQ-BRECON23: Satisfaction with surgery**				
Available (%)	383 (93)	234 (92)	149 (96)	
Mean(s.d.)	64(24)	59(23)	71(25)	<0.000040849
**Subset: age ≥50 years at selection, *n***	386	261	125	
**QLQ-BRECON23: Satisfaction with breast cosmetic**				
Available (%)	377 (98)	256 (98)	121 (97)	
Mean(s.d.)	51(30)	46(29)	62(29)	<0.000040849
**Subset: T2–T4, *n***	319	182	137	
**Q LQ-BRECON23: Satisfaction with breast cosmetic**				
Available (%)	315 (99)	179 (98)	136 (99)	
Mean(s.d.)	53(29)	44(28)	66(26)	<0.000040849
**QLQ-BRECON23: Satisfaction with surgery**				
Available (%)	291 (91)	161 (88)	130 (95)	
Mean(s.d.)	62(28)	54(28)	72(24)	<0.000040849
**Subset: N0, *n***	480	341	139	
**QLQ-BRECON23: Satisfaction with breast cosmetic**				
Available (%)	475 (99)	337 (99)	138 (99)	
Mean(s.d.)	53(28)	48(27)	64(28)	<0.000040849
**QLQ-BRECON23: Satisfaction with surgery**				
Available (%)	446 (93)	315 (92)	131 (94)	
Mean(s.d.)	63(27)	59(26)	70(26)	<0.000040849
**QLQ-BRECON23: Treatment side effects**				
Available (%)	476 (99)	339 (99)	137 (99)	
Mean(s.d.)	9(18)	6(13)	17(24)	<0.000040849
**Subset: RT–, *n***	501	397	104	
**QLQ-BRECON23: Satisfaction with breast cosmetic**				
Available (%)	497 (99)	393 (99)	104 (100)	
Mean(s.d.)	51(28)	47(27)	63(30)	<0.000040849
**QLQ-BRECON23: Satisfaction with surgery**				
Available (%)	467 (93)	368 (93)	99 (95)	
Mean(s.d.)	62(27)	59(26)	71(27)	<0.000040849
**Subset: RT+, *n***	296	119	177	
**QLQ-BRECON23: Satisfaction with breast cosmetic**				
Available (%)	290 (98)	117 (98)	173 (98)	
Mean(s.d.)	56(27)	45(26)	64(26)	<0.000040849
**QLQ-BRECON23: Satisfaction with surgery**				
Available (%)	276 (93)	108 (91)	168 (95)	
Mean(s.d.)	64(26)	55(27)	70(24)	<0.000040849
**Subset: ET–, *n***	467	324	143	
**QLQ-BRECON23: Satisfaction with breast cosmetic**				
Available (%)	461 (99)	321 (99)	140 (98)	
Mean(s.d.)	51(28)	47(27)	61(29)	<0.000040849
**Subset: ET+, *n***	330	192	138	
**QLQ-BRECON23: Satisfaction with breast cosmetic**				
Available (%)	326 (99)	189 (98)	137 (99)	
Mean(s.d.)	55(28)	47(27)	66(26)	<0.000040849
**QLQ-BRECON23: Satisfaction with surgery**				
Available (%)	309 (94)	177 (92)	132 (96)	
Mean(s.d.)	65(27)	59(27)	72(24)	<0.000040849
**Subset: CT–, *n***	523	373	150	
**QLQ-BRECON23: Satisfaction with breast cosmetic**				
Available (%)	519 (99)	370 (99)	149 (99)	
Mean(s.d.)	50(28)	45(27)	61(28)	<0.000040849
**QLQ-BRECON23: Satisfaction with surgery**				
Available (%)	488 (93)	344 (92)	144 (96)	
Mean(s.d.)	61(27)	57(27)	69(26)	<0.000040849
**Subset: CT+, *n***	274	143	131	
**QLQ-BRECON23: Satisfaction with breast cosmetic**				
Available (%)	268 (98)	140 (98)	128 (98)	
Mean(s.d.)	58(28)	51(27)	66(26)	<0.000040849

In functional and satisfaction scales of the QLQ-BRECON23, higher scores are better. In symptom scales of the QLQ-BRECON23, lower scores are better. The survey was administered in 2015–2016, either 15 years (women selected in 2000), 10 years (women selected in 2005) or 5 years (women selected in 2010) after mastectomy. *P* are based on Wilcoxon rank sum test and are only calculated if there are at least three unique values in each group. Due to the Bonferroni adjustment, only *P* < 0.000040849 are considered significant. CT, chemotherapy; ET, endocrine therapy.

Consistently with BRECON23, the BREAST-Q reported satisfaction with breast after autologous reconstruction was higher in the subset of year 2000 (67 *versus* 52; difference of 15 points), among those receiving radiotherapy (RT+; 67 *versus* 54; 13 points), and those with larger tumour (T2–T4) subset (68 *versus* 54; 14 points).

## Discussion

Breast reconstruction plays a crucial role in restoring physical appearance and enhancing the HRQoL following breast cancer. The reported timing of reconstruction in the current nationwide study was 20% immediate and 80% delayed due to differences in local routines and traditions, as well as availability of plastic surgeons in the six Swedish regions. The oncologic treatment priority at the time of mastectomy may also have played a role^13^. The current study could not identify an impact of reconstruction timing on patient-reported outcomes 5, 10 or 15 years after mastectomy.

Previous research comparing immediate *versus* delayed reconstruction is heterogeneous and methodologically limited. A review article by Heimes *et al*.^4^ summarizes that IBR might have a positive impact on patients’ well-being. The majority of the analysed studies, however, showed that psychosocial outcomes did not differ between the groups of mastectomy alone, IBR and DBR.

A recent population based-cohort study in England with a follow-up time of 12 years^14^ reported that women with abdominal flap BR reported higher levels of satisfaction with breasts than other BR groups (that is, expander/implant, latissimus dorsi flap).

In a larger multicentre prospective study of 1639 immediate and 147 delayed reconstruction patients from 11 hospitals in the USA and Canada by Yoon *et al*.^15^, no significant differences were seen in patient satisfaction or in psychosocial, sexual or physical well-being at a 2-year follow-up survey. This is in line with our study that validates and extrapolates these findings to a long-term perspective (5–15 years).

In the current study, delayed reconstruction was associated with higher levels of satisfaction with nipples compared to immediate reconstruction. This observation could be explained by the fact that patients undergoing delayed reconstruction had experienced a period without nipples and consequently they may exhibit a more positive attitude towards nipple reconstruction. In contrast, in cases of nipple-sparing immediate reconstructions, patients retain a reference memory of their native nipples.

Comparing autologous to implant-based breast reconstructions, the autologous methods, which use the patient’s own tissue, were associated with fewer breast symptoms and higher satisfaction scores as in earlier studies^6,14–16^. This confirms that autologous reconstruction may offer more natural-looking and feeling breasts, contributing to improved well-being. Same pattern was seen in the subgroup analyses based on time passed since mastectomy, satisfaction with breasts was higher in the autologous group compared with the implant-based group both in the BRECON23 and BREAST-Q surveys.

A review by Eltahir *et al*.^16^ found, similarly to the current study, that autologous breast reconstruction outperformed implant-based reconstruction in satisfaction with breasts and physical well-being. A similar pattern was observed in a meta-analysis by Toyserkani *et al*^17^.

The findings of the current study may guide healthcare professionals in tailoring the treatment plans and enhance overall care for breast cancer patients. As described in a study by Frost *et al*.^18^, informed decision-making is of importance to women and will affect satisfaction with outcome, as women reported less satisfaction with information they received about different reconstructions methods and timing of reconstruction.

Our study encompassed the entire eligible population of a country and was assessed over a period of 5, 10 and 15 years. This renders the study particularly relevant as it provides insights into long-term outcomes on a national level including diverse demographics. By including a comprehensive population, using validated questionnaires and conducting analyses for clinically relevant subgroups, and a long follow-up period, the study contributes valuable information that can enhance the understanding for HRQoL after breast reconstruction.

The current study carries certain limitations. The retrospective design of the survey years after the surgery lead to data collection limitations. The generalizability may be limited and the findings may not fully apply outside of Sweden in countries with a different population and different healthcare settings. Like in any comparative study on breast reconstruction, a selection bias in reconstruction timing and methods for reconstruction may have occurred. Self-reported questionnaires may be subject to recall or information biases and inaccuracies. Advances in surgical techniques over time, for example reconstructions in 2005 *versus* 2015, may also influence patient-reported outcomes.

In addition to identifying statistically significant differences between groups, which was done in this study using a conservative Bonferroni approach, it is also important to consider whether differences are clinically significant. If a group difference is not statistically significant, we cannot be certain that any observed difference is not due to random chance. and it is of course inappropriate to discuss whether they are clinically relevant. On the other hand, a statistically significant difference is not necessarily clinically relevant or meaningful to patients. No estimates of what is meaningful to patients are available for the scores in the BREAST-Q or QLQ-BRECON23 and, as no appropriate anchors were available in this data set, such thresholds could not be derived in this study.

This Swedish long-term follow-up survey highlights the importance of breast reconstruction for well-being and quality of life postmastectomy. Across various demographic and treatment subgroups, women undergoing autologous reconstruction consistently reported higher levels of satisfaction with both surgical outcomes and breast appearance. Yet no significant impact of reconstruction timing on the outcomes was observed 5–15 years postmastectomy. These findings provide valuable insights for clinicians and patients, facilitating informed decision-making to optimize outcomes and the quality of care for women undergoing mastectomy.

## Supplementary Material

znae240_Supplementary_Data

## Data Availability

The data supporting findings of this study are available upon reasonable request from the corresponding author.
