# Supplementary material for: Timing and type of breast reconstruction in SweBRO 3: long-term outcomes
Source: Br J Surg. 2024 Sep 24;111(9):znae240. doi: 10.1093/bjs/znae240 (PMC11421470; doi:10.1093/bjs/znae240)
Supplement: znae240_Supplementary_Data [file znae240_supplementary_data.docx]

**TItle**

**Timing and Type of Breast Reconstruction in SweBRO 3: Long-Term Outcomes**

Rojda Gümüscü^1^, Fredrik Wärnberg^1, 2^, Jana de Boniface^3^, Malin Sund^4,5^, Kristina Åhsberg^6^, Emma Hansson7, Folke Folkvaljon^8^ , Dmytro Unukovych^9^, and Maria Mani^1,10^

1. Department of Surgical Sciences, Uppsala University, Uppsala, Sweden
2. Department of Surgery, Sahlgrenska University Hospital, Institute of Clinical Sciences, Sahlgrenska Academy at Gothenburg University, Gothenburg
3. Department of Surgery, Capio S:t Göran’s Hospital, Stockholm, Sweden, Department of Medicine and Surgery Karolinska Intitutet, Stockholm, Sweden
4. Department of Surgery and Perioperative Sciences, Umeå University, Umeå, Sweden
5. Department of Surgery, University of Helsinki and Helsinki University Hospital, Finland
6. Department of Surgery, Halland Hospital, Halmstad and Department of Clinical Sciences, Lund University, Lund, Sweden
7. Department of Plastic surgery, Institute of Clinical Sciences, Sahlgrenska Academy, University of Gothenburg and Region Västra Götaland, Sahlgrenska University Hospital, Department of Plastic and Reconstructive Surgery, Gothenburg, Sweden
8. Sveastat AB, Sweden.
9. Department of Plastic and Craniofacial Surgery, Karolinska University Hospital, Stockholm, Sweden
10. ,Department of Plastic and Reconstructive Surgery, Uppsala University Hospital, Uppsala, Sweden

Correspondence:

Rojda Gümüscü MD,

Department of Plastic and Reconstructive Surgery

Uppsala University hospital 751 85 Uppsala, Sweden

E-mail: rojda.gumuscu@surgsci.uu.se

Supplementary Materials - Index

| **Supplementary Methods n/a** |  |
| --- | --- |
| Detail | *pag. X* |
| Detail | *pag. Y* |
| **Supplementary Results n/a** |  |
| Detail | *pag. X* |
| Detail | *pag. Y* |
| **Supplementary Appendixes n/a** |  |
| Detail | *pag. X* |
| Detail | *pag. Y* |
| **Supplementary Figures and Tables** |  |
| Supplementary figure 1 | *pag. 6* |
| Supplementary table 1  Supplementary table 2 | *pag. 7*  *pag. 8* |
| **References n/a** | *pag. Z* |
|  |  |

**Supplementary Methods n/a**

**Supplementary Results n/a**

**Supplementary Appendixes n/a**

**Supplementary Figures and Tables**


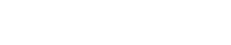


Overall alpha

for planned analyses

*α*

= 0.05


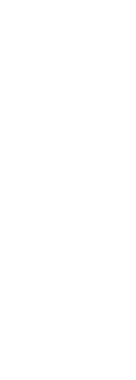


EORTC

QLQ

-

BRECON23

Assigned alpha:

0.05/4 = 0.0125

•

Overall and in

16

subgroups

*

•

2

treatment

comparisons

•

9

domains

Number of tests:

1

7

× 2 × 9

= 306

Significance level

in each test:

0.0125

/

306

<

4.0849

×

10

-

5


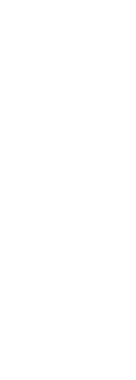


BREAST

-

Q

reconstruction

(

post

-

operative)

Assigned alpha:

0.05/4 = 0.0125

•

Overall and in

16

subgroups

*

•

2

treatment

comparisons

•

1

0

domains

Number of tests:

1

7

× 2 × 1

0

=

340

Significance level

in each test:

0.0125

/

340

<

3.6

764

×

10

-

5


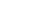


1

/

4


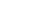


4

/

1


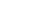


/

4

1


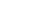


1

4

/


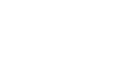


EORTC QLQ

-

C30

Assigned alpha:

0.05/4 = 0.0125


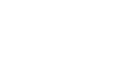


EORTC QLQ

-

BR23

Assigned alpha:

0.05/4 = 0.0125

Supplementary figure 1. Alpha spending across hypothesis tests.

* Prespecified subgroups were analyzed according to selection year (2000, 2005, 2010), age at selection (<50, ≥50 years), tumor stage (T0/Tis, T1, T2-T4), nodal status (N0/N+), adjuvant radiotherapy (yes, no), adjuvant endocrine therapy (yes, no), adjuvant chemotherapy (yes, no).

**Supplementary Figures and Tables (cont.)**

Supplementary table 1. Domains in the EORTC QLQ-BRECON23 and BREAST-Q

|  | **No. of questions** | **Directionality** |
| --- | --- | --- |
| **EORTC QLQ-BRECON23** |  |  |
| **Functional and satisfaction scales** |  |  |
| Sexual functioning | 4 | Higher is better |
| Satisfaction with breast cosmetic | 6 | Higher is better |
| Satisfaction with nipple cosmetic | 2 | Higher is better |
| Satisfaction with surgery | 3 | Higher is better |
| Satisfaction with donor scars | 1 | Higher is better |
| Preserve / reconstruct nipple | 1 | Higher is better |
| **Symptoms scales/items** |  |  |
| Treatment side effects | 3 | Lower is better |
| Donor site symptoms | 2 | Lower is better |
| Loss of nipple | 1 | Lower is better |
| **BREAST-Q reconstruction post-operative module** (version 1.0) |  |  |
| **Satisfaction scales*** |  |  |
| Satisfaction with breasts | 16 | Higher is better |
| Satisfaction with nipples | 5 | Higher is better |
| Satisfaction with information | 15 | Higher is better |
| Satisfaction with surgeon | 12 | Higher is better |
| Satisfaction with medical staff | 7 | Higher is better |
| Satisfaction with office staff | 7 | Higher is better |
| **Well-being scales** |  |  |
| Psychosocial well-being | 10 | Higher is better |
| Sexual well-being | 6 | Higher is better |
| Physical well-being: chest | 16 | Higher is better |
| Physical well-being: abdomen | 8 | Higher is better |

* The BREAST-Q reconstruction post-operative module scale “Satisfaction with outcome” was miscoded in the original data and is therefore not included in any analyses.
EORTC European Organisation for Research and Treatment of Cancer, QLQ‑BRECON23 Quality of Life Questionnaire Breast Reconstruction-23

**Supplementary Figures and Tables (cont.)**

Supplementary table 2. Baseline characteristics and demographics, by reconstruction timing or modality

|  | **Reconstruction timing *** | | **Reconstruction modality *** | |
| --- | --- | --- | --- | --- |
|  | **Immediate (%)** | **Delayed (%)** | **Implant-based (%)** | **Autologous (%)** |
| **Total** | 176 (100) | 719 (100) | 516 (100) | 281 (100) |
| **Age at selection, yr **** |  |  |  |  |
| Median (IQR) | 50 (44-54) | 49 (44-56) | 50 (44-56) | 48 (43-54) |
| < 50 | 88 (50) | 363 (50) | 255 (49) | 156 (56) |
| 50-59 | 63 (36) | 246 (34) | 174 (34) | 93 (33) |
| > 59 | 25 (14) | 110 (15) | 87 (17) | 32 (11) |
| **BMI** |  |  |  |  |
| < 25 | 110 (62) | 400 (56) | 324 (63) | 135 (48) |
| 25-30 | 53 (30) | 242 (34) | 148 (29) | 113 (40) |
| > 30 | 11 (6) | 70 (10) | 40 (8) | 31 (11) |
| Missing data | 2 (1) | 7 (1) | 4 (1) | 2 (1) |
| **Marital status** |  |  |  |  |
| Married | 98 (56) | 417 (58) | 293 (57) | 168 (60) |
| Partner | 26 (15) | 107 (15) | 72 (14) | 46 (16) |
| Widow | 9 (5) | 34 (5) | 26 (5) | 11 (4) |
| Single | 33 (19) | 124 (17) | 92 (18) | 45 (16) |
| Other | 10 (6) | 34 (5) | 32 (6) | 9 (3) |
| Missing data | 0 (0) | 3 (0) | 1 (0) | 2 (1) |
| **No. of children** |  |  |  |  |
| 1 | 27 (15) | 132 (18) | 92 (18) | 49 (17) |
| 2 | 88 (50) | 342 (48) | 245 (47) | 145 (52) |
| 3 | 37 (21) | 149 (21) | 105 (20) | 59 (21) |
| 4 or more | 7 (4) | 37 (5) | 31 (6) | 7 (2) |
| 0/Missing data | 17 (10) | 59 (8) | 43 (8) | 21 (7) |
| **Educational level** |  |  |  |  |
| Elementary school | 14 (8) | 97 (13) | 56 (11) | 38 (14) |
| High school | 55 (31) | 271 (38) | 191 (37) | 99 (35) |
| University | 106 (60) | 341 (47) | 263 (51) | 143 (51) |
| Missing data | 1 (1) | 10 (1) | 6 (1) | 1 (0) |
| **Yearly income, SEK** |  |  |  |  |
| < 100 000 | 3 (2) | 33 (5) | 17 (3) | 14 (5) |
| 100 000 - 250 000 | 34 (19) | 189 (26) | 130 (25) | 64 (23) |
| 250 000 - 400 000 | 65 (37) | 316 (44) | 213 (41) | 133 (47) |
| > 400 000 | 65 (37) | 142 (20) | 123 (24) | 63 (22) |
| Missing data | 9 (5) | 39 (5) | 33 (6) | 7 (2) |
| **Smoking status** |  |  |  |  |
| Non-smoker | 84 (48) | 297 (41) | 223 (43) | 123 (44) |
| Former smoker | 75 (43) | 338 (47) | 230 (45) | 134 (48) |
| Current smoker | 13 (7) | 65 (9) | 51 (10) | 19 (7) |
| Missing data | 4 (2) | 19 (3) | 12 (2) | 5 (2) |
| **T stage** |  |  |  |  |
| T0/Tis | 52 (30) | 92 (13) | 98 (19) | 32 (11) |
| T1 | 53 (30) | 226 (31) | 169 (33) | 79 (28) |
| T2 | 39 (22) | 242 (34) | 147 (28) | 109 (39) |
| T3 | 13 (7) | 56 (8) | 33 (6) | 26 (9) |
| T4 | 0 (0) | 4 (1) | 2 (0) | 2 (1) |
| Tx/Missing data | 19 (11) | 99 (14) | 67 (13) | 33 (12) |
| **N stage** |  |  |  |  |
| N0 | 130 (74) | 403 (56) | 341 (66) | 139 (49) |
| N+ | 18 (10) | 115 (16) | 49 (9) | 70 (25) |
| Nx/Missing data | 28 (16) | 201 (28) | 126 (24) | 72 (26) |
| **M stage** |  |  |  |  |
| Mx/M0 | 173 (98) | 620 (86) | 453 (88) | 254 (90) |
| M1 | 0 (0) | 0 (0) | 0 (0) | 0 (0) |
| Missing data | 3 (2) | 99 (14) | 63 (12) | 27 (10) |
| **Neoadjuvant therapy ***** |  |  |  |  |
| No neoadjuvant therapy | 153 (87) | 575 (80) | 441 (85) | 214 (76) |
| Received neoadjuvant therapy | 8 (5) | 63 (9) | 20 (4) | 41 (15) |
| Missing data or not collected in registry | 15 (9) | 81 (11) | 55 (11) | 26 (9) |
| **Post-operative radiotherapy** |  |  |  |  |
| No post-operative radiotherapy | 123 (70) | 433 (60) | 397 (77) | 104 (37) |
| Received post-operative radiotherapy | 53 (30) | 286 (40) | 119 (23) | 177 (63) |
| **Post-operative endocrine therapy** |  |  |  |  |
| No post-operative endocrine therapy | 133 (76) | 393 (55) | 324 (63) | 143 (51) |
| Received post-operative endocrine therapy | 43 (24) | 326 (45) | 192 (37) | 138 (49) |
| **Post-operative chemotherapy** |  |  |  |  |
| No post-operative chemotherapy | 121 (69) | 466 (65) | 373 (72) | 150 (53) |
| Received post-operative chemotherapy | 55 (31) | 253 (35) | 143 (28) | 131 (47) |
|  |  |  |  |  |

IQR = Interquartile range, BMI = Body mass index

* Reconstruction timing and modality are based on self-reported information.

** Selection was done on year of diagnosis in all regions except for the "South" region in which women were selected based on year of mastectomy.

*** This information was not collected in all regions prior to 2010.

**References n/a**
